# Improving the Effects of Mulberry Leaves and Neochlorogenic Acid on Glucotoxicity-Induced Hepatic Steatosis in High Fat Diet Treated db/db Mice

**DOI:** 10.1021/acs.jafc.3c09033

**Published:** 2024-03-15

**Authors:** Ming-Chang Tsai, Chi-Chih Wang, I-Ning Tsai, Meng-Hsun Yu, Mon-Yuan Yang, Yi-Ju Lee, Kuei-Chuan Chan, Chau-Jong Wang

**Affiliations:** † Division of Gastroenterology and Hepatology, Department of Internal Medicine, Chung Shan Medical University Hospital, Taichung 402, Taiwan; ‡ School of Medical, Chung Shan Medical University, Taichung 402, Taiwan; § Institute of Medicine, Chung Shan Medical University, Taichung 402, Taiwan; ∥ Department of Nutrition, Chung Shan Medical University, No. 110, Section 1, Jianguo North Road, Taichung 402, Taiwan; ⊥ Department of Health Diet and Industry Management, Chung Shan Medical University, Taichung 402, Taiwan; # Department of Pathology, Chung Shan Medical University Hospital, Taichung 402, Taiwan; ∇ School of Medical, Chung Shan Medical University, Taichung 402, Taiwan; ○ Department of Internal Medicine, Chung-Shan Medical University Hospital, No. 110, Section 1, Jianguo North Road, Taichung 402 and Taiwan; ◆ Department of Medical Research, Chung Shan Medical University Hospital, Taichung 402, Taiwan

**Keywords:** antioxidant activities, diabetes mellites, mulberry leaves, neochlorogenic acid, nonalcoholic fatty liver disease

## Abstract

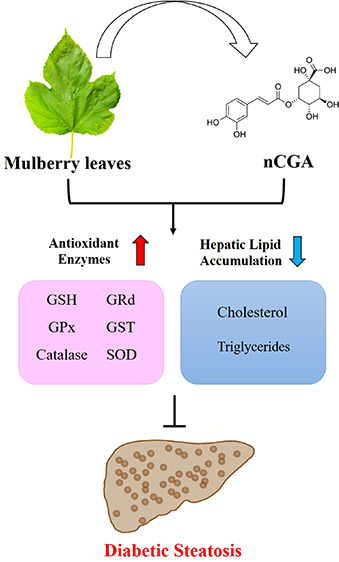

There are many complications of type 2 diabetes mellitus. Nonalcoholic fatty liver disease (NAFLD) and nonalcoholic steatohepatitis (NASH) are two complications related to the increased lipid accumulation in the liver. Previous studies have shown that mulberry leaf water extract (MLE) has the effect of lowering lipid levels in peripheral blood, inhibiting the expression of fatty acid synthase (FASN) and increasing the activity of liver antioxidant enzymes superoxide dismutase (SOD) and catalase. Our study aimed to investigate the role of MLE and its main component, neochlorogenic acid (nCGA), in reducing serum lipid profiles, decreasing lipid deposition in the liver, and improving steatohepatitis levels. We evaluated the antioxidant activity including glutathione (GSH), glutathione reductase (GRd), glutathione peroxidase (GPx), glutathione S-transferase (GST), and superoxide dismutase (SOD), and catalase was tested in mice fed with MLE and nCGA. The results showed a serum lipid profile, and fatty liver scores were significantly increased in the HFD group compared to the db/m and db mice groups, while liver antioxidant activity significantly decreased in the HFD group. When fed with HFD + MLE or nCGA, there was a significant improvement in serum lipid profiles, liver fatty deposition conditions, steatohepatitis levels, and liver antioxidant activity compared to the HFD group. Although MLE and nCGA do not directly affect the blood sugar level of db/db mice, they do regulate abnormalities in lipid metabolism. These results demonstrate the potential of MLE/nCGA as a treatment against glucotoxicity-induced diabetic fatty liver disease in animal models.

## Introduction

The complication of diabetes mellites (DM) arises from cell damage induced by oxidative stress or glycogenesis due to high serum sugar levels and could lead to nonalcoholic fatty liver disease (NAFLD), steatohepatitis, diabetic kidney disease, diabetic neuropathy, major vascular disease, retinopathy, and peripheral vascular disease. The status of DM can also impair the healing ability, especially in the distal part of the extremities. By improving the level of serum glucose, we can delay or even prevent the occurrence of these complications. Dysregulation of lipogenesis, which increases lipid accumulation in the liver, can further lead to insulin resistance or be exacerbated by high serum glucose levels. Therefore, NAFLD and nonalcoholic steatohepatitis (NASH) are correlated with type 2 diabetes mellitus (T2DM), obesity, insulin resistance, oxidative stress, and inflammation. NAFLD can be considered an early predictor of DM.^1^

Insulin resistance, which develops hypertriglyceridemia, decreases the serum level of high-density lipoprotein (HDL) and increases the serum levels of low-density lipoprotein (LDL) and very low-density lipoprotein (VLDL). These results from insulin resistance increasing the degradation of adipose tissue and then producing a large amount of free fatty acid, which raises the level of serum triglycerides (TGs) and apolipoprotein B in the liver and synthesizes large amounts of VLDL. This links NAFLD to atherogenic dyslipidemia and the risk of cardiovascular disease.

The chemical compositions of mulberry leaves include rutin, quercetin, adenine, choline, chlorogenic acid (CA), neochlorogenic acid (nCGA), cryptochlorogenic acid (CCA), inokosterone, stigmasterol, lupeol, acetic acid, propionic acid, butyric acid, glutamic acid, aspartic acid, alanine, bergapten, and umbelliferone.^2^ CA and its isomers, nCGA and CCA, constitute 78% of the polyphenol content.^3^ Existing studies indicate the potential of nCGA in offering antioxidant and hepatoprotective benefits, particularly under specific environmental conditions. These properties of nCGA have been linked to a reduction in kidney fibrosis.^4−6^ Moreover, research has identified nCGA’s role in cancer therapy, where it is found to induce apoptosis and inhibit the proliferation of cancer cells.^7^ Flavonoids in mulberry leaves have antiviral and anti-inflammatory effects and possess antioxidant capacity several times higher than those of vitamins C and E, which helps prevent LDL oxidation, inhibits heart disease caused by atherosclerosis, prevents heart disease, and has anticancer effects.^8^

Nitrogen-containing glycoalkaloids from mulberry leaves are active ingredients in Chinese herbal medicine and can inhibit the activity of glucose-producing enzymes to regulate blood sugar. After identification, it was found that its sugar group is deoxynojirimycin.^9^ Previous research has indicated that the flavonoids in mulberry leaves exhibit antioxidant^10^ and hepatoprotective properties.^11^ Additionally, flavonoids from *Morus alba* L. (*M. alba*) have demonstrated significant antiviral, antibacterial, and anti-inflammatory effects in both laboratory and animal studies.^12^ MLE also are recognized for their crucial roles in reducing blood glucose, lipid levels, and free fatty acids.^13^ Our group previously investigated that Mulberry Leaf Extract (MLE) and neochlorogenic acid (nCGA) effectively reduced liver fat accumulation. This is achieved by inhibiting the SREBP-1/FASN and SREBP-2/HMG-CoAR pathways, and by activating the PPARα/CPT-1 pathway.^14^ Furthermore, MLE has also been shown to inhibit the proliferation of hepatocellular carcinoma cells by suppressing the production of IL-6 and TNF-α from adipocytes.^15^

The CA in mulberry leaves is a powerful antioxidant that protects cells from damage. In previous experiments with obese animal models, CA significantly reduced the body weight, visceral fat, serum triglycerides, and cholesterol levels of rats.^16^ Mulberry leaves can regulate small GTPase and Akt/NF-B pathway,^17^ affect the cell cycle,^18^ and inhibit the proliferation and migration of vascular smooth muscle cells, and its polyphenol extract can improve atherosclerosis.^19^ CA prevents liver damage and induces apoptosis in liver cancer cells by regulating P53.^20,21^

Regarding the absorption of CA, it is known that the small intestine absorbs one-third, while the large intestine can absorb the remaining two-thirds.^22^ The small intestine utilizes quinic and feruloyl quinic acids to convert them into ferulic and caffeic acids. The conversion of caffeic acid and ferulic acid to dihydroferulic acid is crucial for absorption in the colon.^23^ The mechanism of CA involves promoting the entry of fatty acids into liver cells, mainly by inhibiting hepatic peroxisome proliferator-activated receptor γ (PPARγ). In terms of antisteatosis, CA inhibits the production of reactive oxygen species (ROS) caused by a high-fat diet, reduces inflammation, slows insulin resistance, improves fat accumulation, and decreases weight gain. It also inhibits the expression of PPARγ to prevent fatty degeneration.

CA possesses various properties, including antioxidant, anti-inflammatory, antidiabetic, antiobesity, antibacterial, and antihypertensive effects. Previous studies have indicated that CA can slow body weight gain and reduce glucose absorption in humans. CA inhibits intestinal absorption by suppressing glucose-6-phosphotransferase 1, thus preventing glucose release and lowering blood glucose levels. Reduction in insulin levels and fat accumulation can lead to weight loss.^24^ Additionally, CA can enhance adiponectin and the adiponectin receptor AMP-activated protein kinase (AMPK), while reducing the level of expression of hepatic glucose-6-phosphatase.

CA can inhibit the inflammatory response of interleukin-8 (IL-8) and its mRNA expression.^25,26^ Moreover, CA mainly exerts its anti-inflammatory effect by scavenging ROS and inhibiting IL-8 and IKK/NF-κB activation pathways.^26^ It can also down-regulate the activity of adhesion molecules by activating IL-4, reduce the production of pro-inflammatory factors, and decrease the infiltration and activation of liver cells.^27^ On the other hand, nCGA has the highest content among the polyphenols in mulberry leaves,^3^ and previous studies have demonstrated its ability to inhibit the growth of breast cancer cells.^28^ nCGA exhibits an anticancer effect on human intestinal cancer cell Caco-2 and slows down tumor growth by inhibiting cancer cell proliferation.^29^ Furthermore, nCGA can also protect nerves by reducing inflammatory factors such as IL-1 and TNF-α.^30^

In this study, a model of db/db mice and high-fat feeding was established to induce glucose-induced DM steatosis. The study explored how MLE and its main component nCGA can slow DM steatosis by inhibiting glucolipotoxicity.

## Materials and Methods

### Production of Mulberry Leaf Water Extract (MLE)

We took 100 g of dry mulberry leaves and cut them into pieces. Next, we add 3000 mL of deionized water and boiled them for 60 min. After the mixture cooled, we filtered it using Whatman No. 1 filter paper. Then, we concentrate the filtered solution under high pressure and freeze-dried it in a vacuum to remove water, resulting in a dried extract known as MLE. During the experiment, MLE was weighed as a dry powder, dissolved in water, and administered through a tube at a dosage of 250 mg/kg.^31,32^

nCGA, the main polyphenolic component in MLE, was purchased from Chengdu Alfa Biotechnology Co., Ltd. in Chengdu, China. CGA was dissolved in dimethyl sulfoxide, yielding stock solution with a concentration of 100 mM.^33^

### Components Analysis of MLE

The components of MLE were identified by using simplified methods. First, we used a basic high-performance liquid chromatography (HPLC) setup for initial analysis. This involved a standard flow rate and a mixture of two solvents for separating the components. We primarily focused on detecting phenolic acids.

After HPLC, we used liquid chromatography–mass spectrometry (LC-MS) for more detailed identification of these components. This step provided us with a clearer understanding of the specific substances present in MLE. The full process and results were detailed in one of our previous studies.^3^

We identified several phenolic compounds. From 100 g of mulberry leaf, we obtained about 32 g of a lyophilized, polyphenol-rich MLE powder, equating to a 32% yield. Key components identified through HPLC and LC-MS analysis included small percentages of various phenolic acids and flavonoids, such as protocatechuic acid, neochlorogenic acid (nCGA), chlorogenic acid (CGA), and others, such as cryptochlorogenic acid, nicotiflorin, rutin, isoquercitrin, and astragalin.

We calculated the average daily intake of these compounds for mice. For instance, mice fed a diet with 0.5% MLE consumed approximately 177.4 μg of nCGA and 119.2 μg of CGA daily. This quantification helps in understanding the dosage and its potential impact in the experimental settings.

### Model of Glucotoxicity-Induced Diabetic Steatohepatitis in Experimental Animals

The experimental animals used were male BKS.Cg-Dock 7m + /+ Leprdb/JNarl mice, commonly referred to as db/db mice, aged six-week-old. These mice were purchased from the Experimental Animal Center of the National Academy of Sciences. The mice weighed approximately 20 g and were housed in Chung Shan Medicine University Animal Center (IACUC no. 2025). The animals were maintained under a 12 h light and 12 h dark cycle, with a room temperature of 22 ± 2 °C.

The animal experiment consisted of four groups; the number of mice used for each group was three (*n* = 3):

(a) Normal control group (db/m): This group included three male db/m mice with genes originating from the same parents. They were fed a standard diet.
(b) Diabetic mice control group (db): This group consisted of db/db mice with spontaneous hyperglycemia.
(c) Diabetes complication group (HFD): This group comprised db/db mice that were fed a high-fat diet (HFD; containing 10% lard oil +2% cholesterol) to induce hyperglycemia, hyperlipidemia, and the occurrence of diabetic complications.
(d) MLE (or nCGA) feeding group: In this group, db/db mice were fed the HFD along with 250 mg/kg of MLE (or nCGA).

The induction of DM was assessed based on the criteria of a body weight increase of at least 10% and a significant increase in blood sugar levels exceeding 200 mg/dL after HFD feeding. Fresh feed was provided daily, and the old feed was removed. The mice’s daily food intake and water consumption were recorded throughout the 12 week experimental period.

### Serum Biochemical Parameters Analysis

The blood samples were centrifuged at 3000*g* for 10 min at 4 °C and stored at −80 °C until they are required for biochemical analysis. The concentrations of HbA1c, cholesterol, triglycerides, glucose, HDL-C, LDL-C, AST, and ALT were measured using the following methods: enzymatic colorimetric assays and commercial kits (Randox Laboratories, Ltd., Antrim, UK). Insulin was measured using an ELISA kit (Mercodia AB, Uppsala, Sweden).

### Fatty Liver and Fibrosis Scores

The severity of steatosis and fibrosis is evaluated by a pathologist, and each is classified on a scale from 0 to 4 based on the extent of fat deposition and fibrotic changes observed in histological examinations. For steatosis, grade 0 signifies normal with 0% fat deposition; grade 1 is mild with less than 10%; grade 2 is moderate with 10–33%; grade 3 is moderate–severe with 33–66%; and grade 4 is severe with 66–100% fat deposition. Similarly, for fibrosis, grade 0 indicates normal with no fibrotic changes; grade 1 is mild fibrosis involving less than 10%; grade 2 is moderate with 10–33%; grade 3 is moderate–severe with 33–66%; and grade 4 is severe fibrosis involving 66–100% of the examined tissue.^34^

### Analysis of Antioxidant Activity

Assay methods for antioxidant enzymes were performed according to the previous studies.^35^ The activity of SOD was measured with the modified method proposed by Marklund.^36^ Catalase activity was determined by the method of Abei.^37^ The activities of GSH, GPx, GRd, and GST were determined with a modified Lawrence and Burk method.^38^

### Hematoxylin and Eosin (H&E) Staining

For staining, the tissue slices were first immersed in hematoxylin for 30 s to stain the cell nuclei. Afterward, they were washed with distilled water and subsequently treated with eosin for 2–5 min. When comparing histopathology, the semiquantitative method developed by Jonker et al.^100^ can be employed to assess the degree of liver cell inflammation, lipid degeneration, liver cell necrosis, and bile duct hyperplasia in chronic liver injury. The evaluation scores range from “0” to “4”, with “0” indicating absence, “1” representing trace, “2” indicating slight, “3” representing moderate, and “4” indicating extremely strong.

### Oil-Red O Staining

Oil-red O staining is a common histological staining method used to detect and quantify the presence of lipid droplets in the liver.^39^ Lipid droplets stained with Oil-red O appear as bright red-orange and can be observed under a microscope. The presence and severity of steatosis can be assessed on the basis of the number and size of lipid droplets in the histopathological analysis.

### Statistical Analysis

The results of this study were initially compared using ANOVA (analysis of variance) and further confirmed using Duncan’s multiple range test. Data were analyzed in GraphPad Prism version 8.0.2, and statistical significance was defined as *p* < 0.05.

## Result

### Steatohepatitis of DM Mice

As shown in Table 1 and Table 2, it is evident that liver enzymes (AST and ALT) were significantly higher in the HFD group compared to those in db mice (*p* < 0.05). This finding indicates that HFD feeding can cause liver damage. However, after MLE or nCGA, the levels of AST and ALT levels decreased significantly compared to the HFD-induced group (*p* < 0.05). When comparing db mice with db/m mice, it was observed that although the AST and ALT values in db mice were higher than those of db/m mice, no statistical difference was found between the two groups. This suggests that db mice alone exhibit symptoms of hyperglycemia without further hepatitis. However, steatohepatitis occurred in the HFD group. MLE and nCGA exhibited a protective effect when administered following the HFD group.

**Table 1:** Plasma Biochemical Parameters in HFD-fed db/db Mice Treated with MLE^a^

	db/m	db	HFD	MLE
AST (U/L)	73.67 ± 8.08	66.33 ± 5.77 c	248.67 ± 51.38 ab	155.67 ± 21.94 abc
ALT (U/L)	32.33 ± 1.53	57.67 ± 22.37 c	527.00 ± 21.07 ab	360.00 ± 38.35 abc
Cholesterol (mg/dL)	61.67 ± 14.36	227.67 ± 4.16 ac	462.67 ± 10.21 ab	350.00 ± 33.72 abc
TG (mg/dL)	84.33 ± 19.43	166.67 ± 31.79 ac	337.00 ± 36.01 ab	149.67 ± 18.45 ac
LDL-C (mg/dL)	18.67 ± 3.51	24.33 ± 6.43 c	173.67 ± 7.37 ab	118.67 ± 3.06 abc
HDL-C (mg/dL)	35.00 ± 7.81	112.67 ± 11.50 ac	144.33 ± 10.69 ab	113.67 ± 11.06 ac
HbA1c (%)	3.97 ± 0.12	7.93 ± 0.76 a	9.03 ± 0.76 a	8.73 ± 0.51 a
Insulin (pg/L)	1.02 ± 0.11	5.10 ± 0.21 ac	8.31 ± 1.47 ab	5.38 ± 0.70 ac

a The age-matched heterozygous mice were assigned to the normal diet group (db/m). The db/db mice were divided into two groups: the normal diet group (db) and the high-fat diet group (HFD). Additionally, another group of db/db mice was fed HFD and administered MLE (MLE). Each value is presented as the mean ± SD (*n* = 3/group). Statistical analysis was performed using ANOVA. a, *p* < 0.05 compared with the db/m group; b, *p* < 0.05 compared with the db group; c, *p* < 0.05 compared with the HFD group.

**Table 2:** Plasma Biochemical Parameters in HFD-Fed db/db Mice Treated with nCGA^a^

	db/m	db	HFD	nCGA
AST (U/L)	74.33 ± 9.02	79.00 ± 13.08 c	255.67 ± 46.52 ab	164.33 ± 11.02 abc
ALT (U/L)	36.67 ± 7.37	64.33 ± 21.73 c	561.00 ± 78.46 ab	262.00 ± 26.46 abc
Cholesterol (mg/dL)	72.33 ± 14.36	210.67 ± 32.62 ac	448.00 ± 23.64 ab	171.33 ± 53.52 ac
TG (mg/dL)	79.00 ± 14.42	160.67 ± 36.94 c	341.00 ± 42.51 ab	153.33 ± 31.79 ac
LDL-C (mg/dL)	18.00 ± 4.58	21.33 ± 5.13 c	177.67 ± 8.74 ab	58.67 ± 6.43 abc
HDL-C (mg/dL)	41.67 ± 9.71	106.33 ± 21.78 ac	142.67 ± 12.01 ab	59.33 ± 15.95 bc
HbA1c (%)	4.03 ± 0.15	8.93 ± 0.95 a	9.53 ± 0.57 a	8.80 ± 0.62 a
Insulin (pg/L)	0.97 ± 0.10	5.42 ± 0.40 ac	9.00 ± 0.86 ab	6.26 ± 0.48 ac

a The age-matched heterozygous mice were assigned to the normal diet group (db/m). The db/db mice were divided into two groups: the normal diet group (db) and the high-fat diet group (HFD). Additionally, another group of db/db mice was fed HFD and administered nCGA (nCGA). Each value is presented as the mean ± SD (*n* = 3/group). Statistical analysis was performed using ANOVA. a, *p* < 0.05 compared with the db/m group; b, *p* < 0.05 compared with the db group; c, *p* < 0.05 compared with the HFD group.

### Effects of MLE and nCGA in Serum Lipid Profile of DM Mice

Compared with the db/m and the db mice, the serum CHO and TG concentrations in db mice significantly increased (*p* < 0.05). After HFD administration, the serum CHO and TG levels in the HFD group further increased, especially compared to the db mice (*p* < 0.05). However, the serum CHO/TG level significantly decreased (*p* < 0.05) after administration of MLE or nCGA along with HFD, suggesting that MLE and nCGA can improve the serum lipid profile when fed with HFD (Table 1 and Table 2).

### Effects of MLE and nCGA in Serum Insulin Level and HbA1c of DM Mice

The HbA1c and serum insulin levels are significantly higher in db mice than in the db/m mice (*p* < 0.05). However, the increase in HbA1c level in the HFD group compared to db mice did not reach the level of significance. On the other hand, the serum insulin level significantly increased (*p* < 0.05) in the HFD group compared to db mice. When mice were fed with MLE and nCGA, the HbA1c level only showed a slight decrease, while the serum insulin level significantly decreased compared to that of the HFD group. These results indicate an improvement in the serum insulin level after MLE and nCGA administrations (Table 1 and Table 2).

### Effects of MLE and nCGA in Liver Histology of DM Mice

The liver weight and liver–body weight ratio (liver weight/mice body weight) of mice in the db mice is higher than those of the db/m mice, while the liver weight increased significantly (*p* < 0.05) in the HFD group compared with the db mice. The liver weight in the MLE and nCGA group decreased compared with the HFD group (*p* < 0.05) (Table 3 and Table 4).

**Table 3:** Effects of MLE on the Change of Organ Weight in HFD-Fed db Mice^a^

	db/m	db	HFD	MLE
Liver weight (g)	2.74 ± 0.06	2.97 ± 0.30 c	5.05 ± 0.49 a	4.58 ± 0.15 ab
Liver triglyceride (mg/(g liver weight))	79.07 ± 5.24	133.24 ± 14.96 ac	259.62 ± 15.12 ab	172.79 ± 9.72 abc
Liver cholesterol (mg/(g liver weight))	99.04 ± 7.37	125.05 ± 11.67 c	213.72 ± 21.21 ab	153.13 ± 19.70 ac
Fatty liver score^b^	0 ± 0.00	0.67 ± 0.58 c	4 ± 0.00 ab	2.67 ± 0.58 abc
Fibrosis score^c^	0 ± 0	0 ± 0	0 ± 0	0 ± 0

a The age-matched heterozygous mice were assigned to the normal diet group (db/m). The db/db mice were divided into two groups: the normal diet group (db) and the high-fat diet group (HFD). Additionally, another group of db/db mice was fed HFD and administered MLE group (MLE). Each value is presented as the mean ± SD (*n* = 3/group). Statistical analysis was performed using ANOVA. a, *p* < 0.05 compared with the db/m group; b, *p* < 0.05 compared with the db group; c, *p* < 0.05 compared with the HFD group.

b Fat deposition condition in histology: normal (0%); mild (<10%, 1); moderate (10–33%, 2); moderate–severe (33–66%, 3); severe (66–100%, 4).

c Fibrosis score defined by histology results: normal (0%); mild (<10%, 1); moderate (10–33%, 2); moderate–severe (33–66%, 3); severe (66–100%, 4).

**Table 4:** Effects of nCGA on the Change of Organ Weight in HFD-Fed db Mice^a^

	db/m	db	HFD	nCGA
Liver weight (g)	2.78 ± 0.09	2.87 ± 0.29 c	5.41 ± 0.04 ab	4.24 ± 0.44 ab
Liver triglyceride (mg/(g of liver weight))	77.32 ± 8.12	134.57 ± 12.80 ac	258.69 ± 12.37 ab	164.46 ± 6.06 abc
Liver cholesterol (mg/(g of liver weight))	98.37 ± 7.33	126.38 ± 13.80 c	214.05 ± 16.77 ab	151.80 ± 17.90 ac
Fatty liver score^b^	0 ± 0.00	0.67 ± 0.58 c	4 ± 0.00 ab	2.33 ± 0.58 abc
Fibrosis score^c^	0 ± 0	0 ± 0	0 ± 0	0 ± 0

a The age-matched heterozygous mice were assigned to the normal diet group (db/m). The db/db mice were divided into two groups: the normal diet group (db) and the high-fat diet group (HFD). Additionally, another group of db/db mice was fed HFD and administered nCGA group (nCGA). Each value is presented as the mean ± SD (*n* = 3/group). Statistical analysis was performed using ANOVA. a, *p* < 0.05 compared with the db/m group; b, *p* < 0.05 compared with the db group; c, *p* < 0.05 compared with the HFD group.

b Fat deposition condition in histology: normal (0%); mild (<10%, 1); moderate (10–33%, 2); moderate–severe (33–66%, 3); severe (66–100%, 4).

c Fibrosis score defined by histology results: normal (0%); mild (<10%, 1); moderate (10–33%, 2); moderate–severe (33–66%, 3); severe (66–100%, 4).

**Figure 1:**
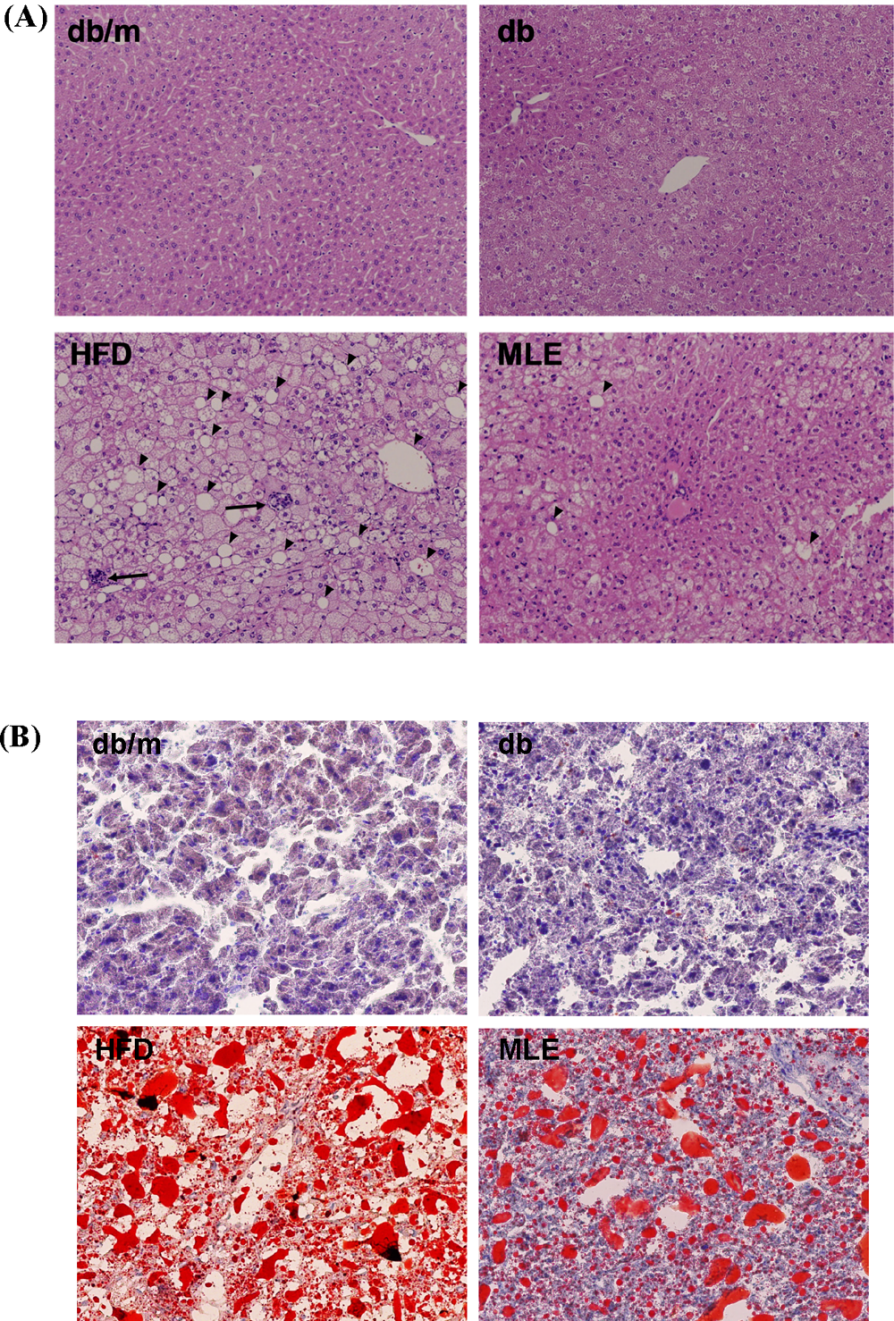
MLE reduction of hepatic steatosis in HFD-induced diabetic mice. Six-week-old male db/db mice fed with HFD and MLE for 12 weeks. Liver frozen sections were stained using H&E (A) and Oil red O (B). The arrow indicates the presence of polymorphonuclear leukocytes. The experimental groups consisted of age-matched heterozygous mice assigned to the normal diet group (db/m), db/db mice divided into two groups (normal diet group (db) and high-fat diet group (HFD)), and a group of db/db mice fed HFD and administered MLE (MLE).

**Figure 2:**
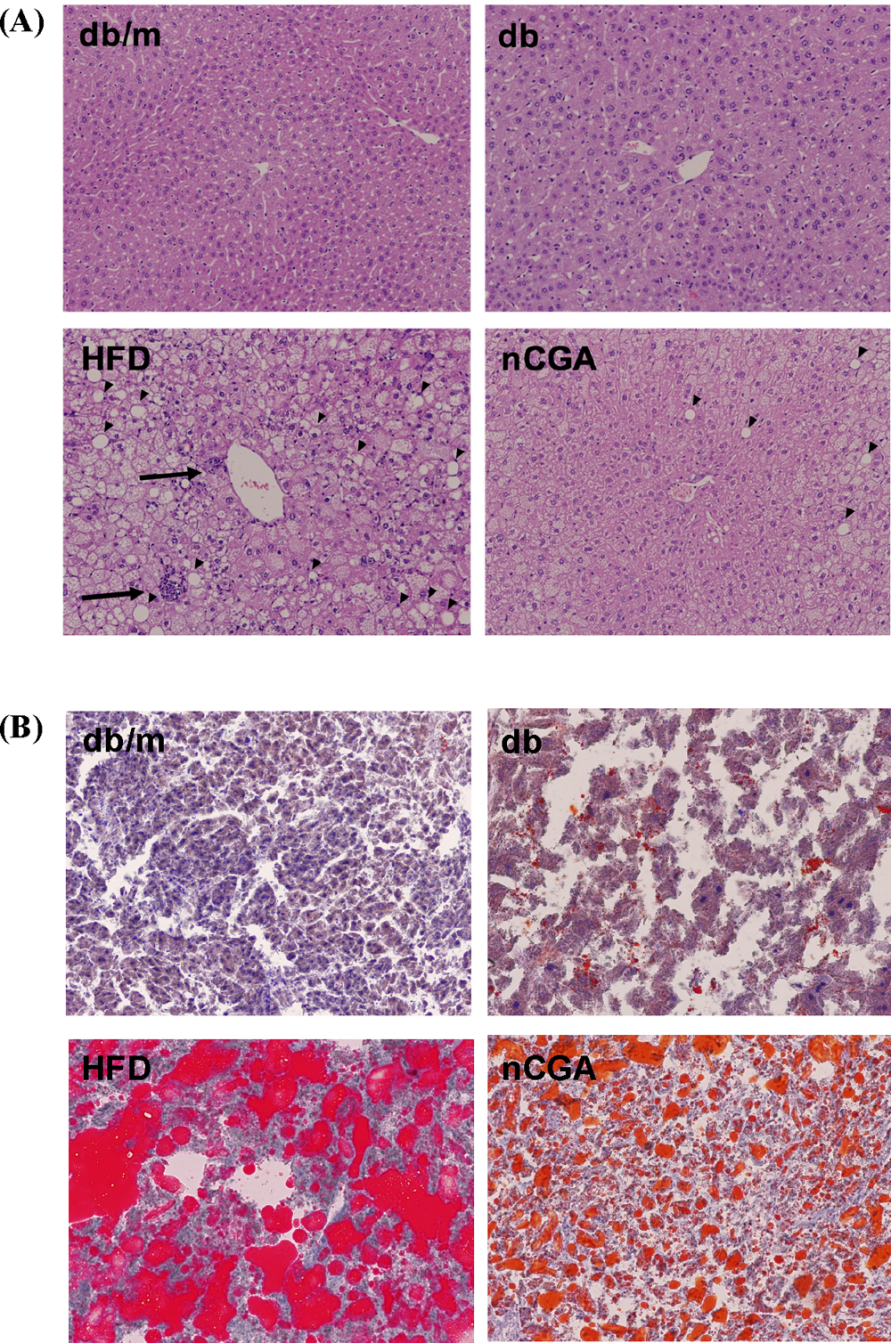
nCGA reduction of hepatic steatosis in HFD-induced diabetic mice. Six-week-old male db/db mice fed with HFD and MLE for 12 weeks. Liver frozen sections were stained using H&E (A) and Oil red O (B). The arrow indicates the presence of polymorphonuclear leukocytes. The experimental groups consisted of age-matched heterozygous mice assigned to the normal diet group (db/m), db/db mice divided into two groups (normal diet group (db) and high-fat diet group (HFD)), and a group of db/db mice fed HFD and administered nCGA (nCGA).

We further analyzed the liver tissue slices to observe changes in lipid accumulation. Numerous vacuoles filled with excess lipids were observed between hepatocytes, indicating that HFD increases excess lipid accumulation in the livers of db/db mice, leading to liver damage. In MLE or nCGA groups, liver tissue sections showed a significant improvement in lipid accumulation with a considerable reduction in the number and size of lipid droplets. These results indicate that MLE and nCGA can reduce lipids in the liver (Figure 1, 2).

### Effects of MLE and nCGA on Liver Antioxidant Enzymes of DM Mice

As shown in Table 5 and Table 6 significant decreases in the activities of antioxidant enzymes including GSH, GPx, GRd, SOD, catalase, and GST are indicate in both the db mice and HFD group compared to the db/m mice. However, in the HFD + MLE or nCGA group, there were significant increases in the activities of antioxidant enzymes compared to the HFD group. This indicates that the supplementation of MLE or nCGA was able to restore the activities of these antioxidant enzymes to levels comparable to those of the normal control group, indicating a potential protective effect against oxidative stress in the liver.

**Table 5:** Effects of MLE on the Antioxidant Enzymes in the Liver of HFD-Fed db Mice^a^

b	db/m	db	HFD	MLE
GSH (μg/(mg of protein))	280.71 ± 13.34	144.27 ± 28.55 a	104.41 ± 29.08 a	252.57 ± 19.94 bc
GPx (nmol of NADPH/min/(mg of protein))	0.051 ± 0.015	0.041 ± 0.008 a	0.032 ± 0.008 a	0.045 ± 0.005 bc
GRd (nmol of NADPH/min/(mg of protein))	0.050 ± 0.009	0.039 ± 0.008 c	0.020 ± 0.008 ab	0.040 ± 0.007 c
GST (nmol/min/(mg of protein))	1.71 ± 0.08	1.52 ± 0.07 a	1.24 ± 0.03 ab	1.66 ± 0.13 c
SOD (U/(mg of protein))	297.33 ± 6.11	215.83 ± 46.13 ac	147.83 ± 17.04 ab	216.00 ± 19.64 ac
Catalase (U/(mg of protein))	375.13 ± 30.39	258.62 ± 34.47 ac	189.49 ± 20.81 ab	296.43 ± 31.02 ac

a The age-matched heterozygous mice were assigned to the normal diet group (db/m). The db/db mice were divided into two groups: the normal diet group (db) and the high-fat diet group (HFD). Additionally, another group of db/db mice was fed HFD and administered MLE (MLE). Each value is presented as the mean ± SD (*n* = 3/group). Statistical analysis was performed using ANOVA. a, *p* < 0.05 compared with the db/m group; b, *p* < 0.05 compared with the db group; c, *p* < 0.05 compared with the HFD group.

b GSH, glutathione; GPx, glutathione peroxidase; GRd, glutathione reductase; GST, glutathione S transferase; SOD, superoxide dismutase.

**Table 6:** Effects of nCGA on the Antioxidant Enzymes in the Liver of HFD-Fed db Mice^a^

b	db/m	db	HFD	nCGA
GSH (μg/(mg of protein))	302.47 ± 13.80	147.21 ± 20.88 a	116.46 ± 23.41 a	255.65 ± 11.60 abc
GPx (nmol of NADPH/min/(mg of protein))	0.059 ± 0.007	0.033 ± 0.009 a	0.020 ± 0.007 a	0.045 ± 0.008 ac
GRd (nmol of NADPH/min/(mg of protein))	0.053 ± 0.004	0.038 ± 0.006 ac	0.019 ± 0.007 ab	0.043 ± 0.004 ac
GST (nmol/min/(mg of protein))	1.74 ± 0.10	1.45 ± 0.11 a	1.23 ± 0.03 ab	1.65 ± 0.11ac
SOD (U/(mg of protein))	293.00 ± 11.00	189.17 ± 27.44 ac	152.17 ± 14.01 ab	215.33 ± 9.44 ac
Catalase (U/(mg of protein))	371.79 ± 10.09	251.95 ± 24.54 ac	192.82 ± 24.81 ab	298.43 ± 23.20 ac

a The age-matched heterozygous mice were assigned to the normal diet group (db/m). The db/db mice were divided into two groups: the normal diet group (db) and the high-fat diet group (HFD). Additionally, another group of db/db mice was fed HFD and administered nCGA (nCGA). Each value is presented as the mean ± SD (*n* = 3/group). Statistical analysis was performed using ANOVA. a, *p* < 0.05 compared with the db/m group; b, *p* < 0.05 compared with the db group; c, *p* < 0.05 compared with the HFD group.

b GSH, glutathione; GPx, glutathione peroxidase; GRd, glutathione reductase; GST, glutathione S transferase; SOD, superoxide dismutase.

## Discussion

There are several essential features and symptoms that characterize T2DM, including obesity, inflammation, insulin resistance, and hyperinsulinemia, which can even be recognized by artificial intelligence.^40,41^ An appropriate experimental animal model is crucial for understanding and analyzing the functional changes of related tissues, insulin sensitivity, insulin hormone production, β-cell proliferation, insulin resistance, and overall glucose homeostasis. In the study of diabetic diseases, the STZ-induced diabetes model is indeed one of the most widely used experimental animal models.^42,43^ STZ selectively destroys pancreatic β-cells, but the effects of STZ can vary depending on factors such as the type of experimental animals, their breed, size, weight, fasting state, and route of administration, etc. Consequently, the diabetes pattern induced by STZ is not as stable as the diabetes model caused by a gene mutation. Currently, there are known obesity genes (obese, ob),^44^ diabetes genes (diabetic, db),^45^ and fat genes (fatty, fa). Common mutations include ob/ob mice, db/db mice, and obese Zucker (fa/fa) rats.^46^ Both ob/ob and db/db mice exhibit symptoms of diabetes, such as hyperglycemia, hyperlipidemia, and obesity, making them excellent animal models for studying diabetes. However, it is important to note that diabetes is a chronic disease that can lead to serious complications. Current ob/ob or db/db mice models only show symptoms of diabetes but do not guarantee the presence of diabetic complications.

Our study results showed diabetic steatohepatitis was successfully induced in db/db mice combined with 12 consecutive weeks of HFD stimulation, including symptoms such as diabetes, hyperglycemia, hyperlipidemia, and obesity. The experiment confirmed that when db/db mice were fed a normal diet for 12 weeks, apart from higher blood AST, ALT, and TC levels and liver weight compared to normal control C57BL/6 mice (db/m), there was no significant excessive lipid accumulation observed in the liver. However, in the HFD group, the blood levels of AST, ALT, TG, and TC were higher than those of the db/m mice and the liver weight was also significantly increased compared to that of the db/m mice. The Oil-red O staining revealed substantial accumulation of lipids and PMN in the HFD group, indicating that the HFD not only successfully induced diabetic fatty liver but also induced steatohepatitis. These findings are consistent with previous evidence,^47^ which indicated that the liver TG content of db/db mice is similar to C57BL/6 mice when fed a normal diet, but significantly higher after being fed a HFD in db/db mice.

A previous study demonstrated that mulberry leaves (MLP, mulberry leaf powder) and MLE have the effect of reducing hyperglycemia, but were not observed in our study.^48,49^ We speculate that this difference may be due to the influence of gene mutation or the possibility that the animal model we established this time is not suitable for investigating the hypoglycemic effect of mulberry leaves. Although the effects of MLE and nCGA on polysaccharide breakdown had no effect on db/db mice, the results of this study confirmed that MLE has the ability to inhibit the development of a diabetic fatty liver. MLE has been reported to reduce lipid levels in peripheral blood,^50^ which is highly consistent with our results. We speculated that MLE prevents the formation of a diabetic fatty liver by reducing blood cholesterol levels. Previous studies have demonstrated that MLE can prevent fatty liver formation by inhibiting the expression of the liver fatty acid synthesis protein FASN and increasing the activity of liver antioxidant enzymes, such as SOD and catalase.^35,51^

The antifat accumulation effects are primarily linked to the antioxidant properties of the compounds. Antioxidants help reduce oxidative stress and inflammation, which are key factors in the development of obesity and excessive fat accumulation. Additionally, some of these compounds may directly interfere with the processes involved in fat cell differentiation and lipid storage, further contributing to their antifat accumulation effects. In previous examined MLE have been studied for their potential in reducing fat accumulation, especially in the context of obesity.^52^ The active compounds in mulberry leaves may inhibit the differentiation of fat cells (adipocytes) and reduce the level of accumulation of fat within these cells. This effect can be enhanced through certain processes like bioconversion, which may increase the bioavailability and efficacy of the active compounds; thus, both MLE and nCGA possess the ability to alleviate the severity of steatohepatitis and diabetic fatty liver.

Chlorogenic acid, the ester of caffeic acid and quinic acid, is known for its diverse biological effects, including antioxidant, anti-inflammatory, hepatoprotective, antimicrobial, cardioprotective, anticarcinogenic, neuroprotective, antiobesity, and antidiabetic properties.^4^ Previous studies have demonstrated that caffeic acid can reduce oxidative stress and potentially prevent cellular damage.^53^ Similarly, quinic acid is thought to prevent the oxidation of free radicals and inhibit the release of inflammatory mediators like NFκB, TNFα, and NO, all of which play roles in inflammation and cellular stress.^54^ Therefore, it can be hypothesized that chlorogenic acid and its isomer, such as nCGA, may exert their effects not only directly but also through hydrolysis into caffeic acid and quinic acid within the body.

## Conclusion

MLE and nCGA exhibit promise in the prevention and treatment of the diabetic fatty liver. They demonstrated the ability to effectively enhance the activity of liver antioxidant enzymes, consequently inhibiting lipogenesis. However, further studies in the future are required to investigate the molecular mechanisms underlying the inhibitory effects of MLE and nCGA and to explore the effectiveness of other potential compounds in the prevention and treatment of diabetic fatty liver.
